# Dynamics of ultrafast phase transitions in MgF_2_ triggered by laser-induced THz coherent phonons

**DOI:** 10.1038/s41598-022-09815-4

**Published:** 2022-04-22

**Authors:** Evgenii Mareev, Fedor Potemkin

**Affiliations:** 1 Faculty of Physics, M. V. Lomonosov Moscow State University, Moscow, Russia 119991; 2 Institute of Photon Technologies of Federal Scientific Research Centre “Crystallography and Photonics” of Russian Academy of Sciences, Troitsk, Russia 108840

**Keywords:** Nonlinear optics, Ultrafast photonics, Phase transitions and critical phenomena

## Abstract

The advent of free-electron lasers opens new routes for experimental high-pressure physics, which allows studying dynamics of condensed matter with femtosecond resolution. A rapid compression, that can be caused by laser-induced shock impact, leads to the cascade of high-pressure phase transitions. Despite many decades of study, a complete understanding of the lattice response to such a compression remains elusive. Moreover, in the dynamical case (in contrast to quasi-static loading) the thresholds of phase transitions can change significantly. Using the third harmonic pump–probe technique combined with molecular dynamics to simulate the terahertz (THz) spectrum, we revealed the dynamics of ultrafast laser-induced phase transitions in MgF_2_ in all-optical experiment. Tight focusing of femtosecond laser pulse into the transparent medium leads to the generation of sub-TPa shock waves and THz coherent phonons. The laser-induced shock wave propagation drastically displaces atoms in the lattice, which leads to phase transitions. We registered a cascade of ultrafast laser-induced phase transitions (P42/mnm ⇒ Pa-3  ⇒ Pnam) in magnesium fluoride as a change in the spectrum of coherent phonons. The phase transition has the characteristic time of 5–10 ps, and the lifetime of each phase is on the order of 40–60 ps. In addition, phonon density of states, simulated by molecular dynamics, together with third-harmonic time-resolved spectra prove that laser-excited phonons in a bulk of dielectrics are generated by displacive excitation (DECP) mechanism in plasma mediated conditions.

## Introduction

With the advent of femtosecond ultrashort laser systems, they have become widespread in fundamental science and the practical field. The femtosecond duration of the laser pulse allows to strongly localize the effect, thereby opening the way for new femtotechnologies for three-dimensional micro- and nano-processing of the bulk of various materials, competing with expensive and proven methods of electron lithography^1^. Furthermore, tight focusing of femtosecond low-energy (up to 10 μJ) laser radiation into the volume of a condensed medium creates the extreme states of matter that occur in stars and can thus be observed in laboratory conditions^2^.

The evolution of the extreme state of a matter created by laser radiation is a cascade of complex processes, ranging from the formation of a non-equilibrium laser microplasma, its thermalization with the transfer of energy from the electronic subsystem to the ion core, generation of shock waves, and the formation of residual micromodifications^3^. These processes occur on a wide time scale from femtoseconds to microseconds, and the final state strongly depends on the features of structural rearrangements in the substance at each of the time stages^4,5^. Understanding microscopic processes in materials and devices that can be switched by light requires experimental access to dynamics on nanometer length and femtosecond time scales that enable new paths to material processing^2^, phase transitions^6,7^, and material properties manipulations^8,9^. The extreme impact of the laser pulse on the matter induces phase transitions that change its structural and physical properties^4,10^. The structural properties of matter are primarily dependent on the symmetry. The symmetry change that could be produced under phase transition opens the way to manipulate material properties on ultrafast (up to sub-ps and fs) timescale. Thereby, it is essential to characterize the dynamics of phase transitions^11^.

Traditionally, changes in the structure of a substance can be diagnosed using X-ray methods^12–14^. However, the sub-picosecond time resolution is only possible with mega-science facilities such as synchrotron radiation sources or free-electron lasers^15^. The development of optical methods (such as Raman scattering^16,17^ or phonon spectroscopy^7,18,19^ for registering structural changes in matter is an essential direction in developing express methods of structural diagnosis with high spatial and temporal resolution. This method can be used and spread more widely, making the developed approach highly relevant to condensed matter physics^11,20^. The generation of coherent phonons upon laser excitation is an important indicator of changes in the structure of a substance^11,21^. The registration of phonon vibrations spectrum in time sheds light on the initiated fast phase transitions. It can provide essential information on the formation of new, stationary and non-stationary phases of matter, which significantly expands the understanding of phase diagrams of matter^11^. In this Letter, we have retrieved the dynamics of ultrafast phase transitions in MgF_2_ from the change of phonon spectrum probed by pump-probe third-harmonic generation technique. The following study will help better understand ultrafast processes during energy transfer from electron plasma to lattice under high deposited energy densities.

## Results and discussion

**Figure 1:**
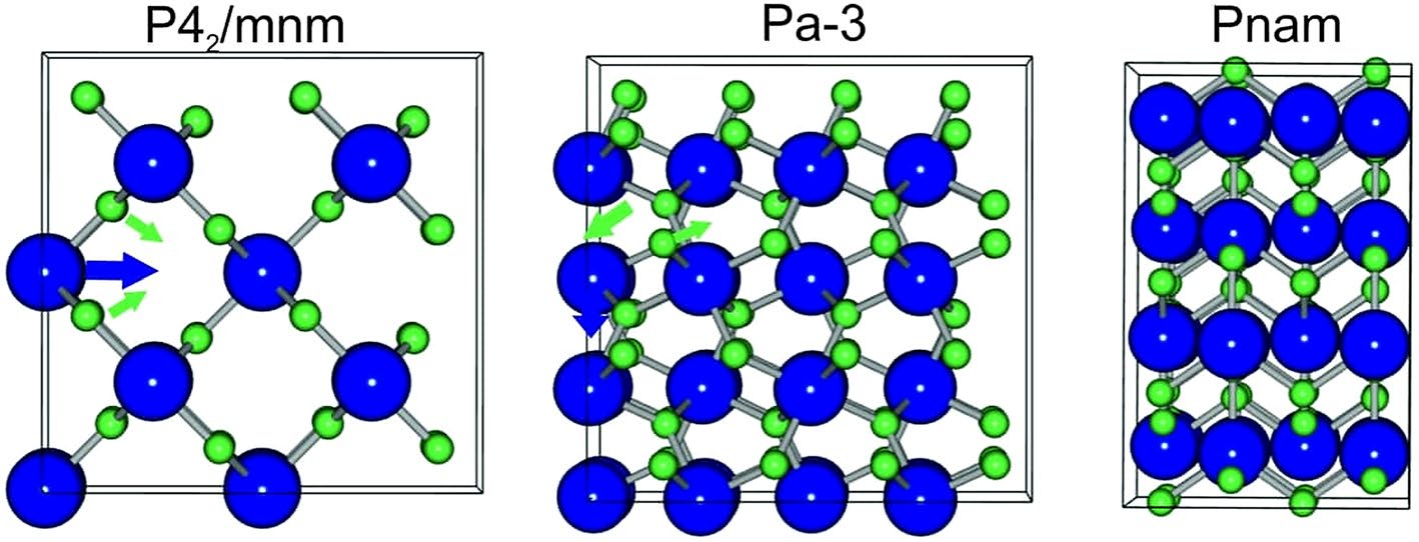
Atoms position inside the MgF_2_ lattice in different phases. Arrows indicate the atom shifts during phase transitions. Mg is imaged as a blue sphere and F as a green.

At ambient conditions, MgF_2_ is in a tetragonal rutile type structure with space group *P42/mnm*, which is the same as TiO_2_ and SiO_2_^22^. Under pressure of 9.1 GPa, it transforms to the orthorombic CaCl2-type phase with a space group *Pnnm*^23^*.* In MgF_2_ crystal, the phase transition at room temperature occurs under static pressure loading^22^. Under further compression, MgF_2_ transforms to the modified fluoride structure (PdF_2_ type, with space group Pa-3) at 14 GPa, and then the cotunnite structure (α-PbCl2 type, with space group Pnam) at 35 GPa^22^. The lattice structure of MgF2 is presented in Fig. 1. It is essential to mention that under high pressure, the phase transition should occur in a cascade manner, as it is demonstrated for the shock-wave induced phase transitions in Si: α-diamond ⇒ β-Sn ⇒ Imma ⇒ simple hex^24–26^. The possible paths of atom movement during solid-to-solid phase transitions are also marked in Fig. 1.

**Figure 2:**
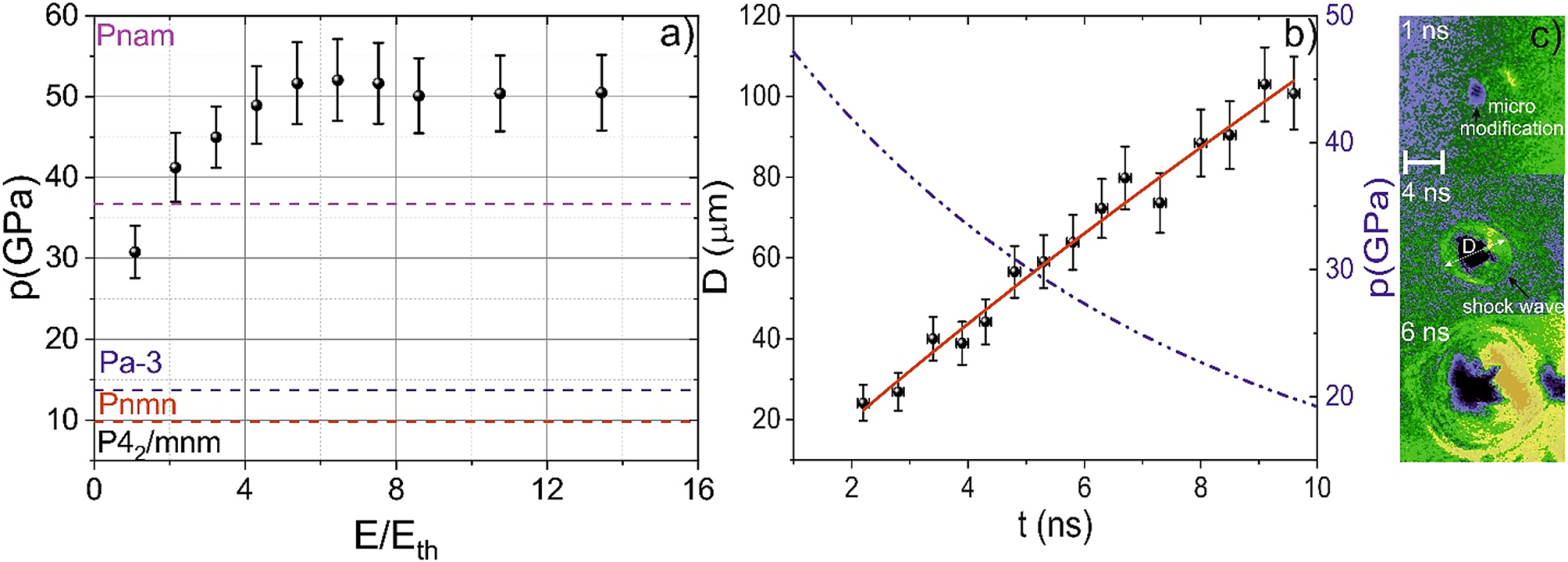
(**a**) Pressure at the shock front as a function of the laser energy normalized to the threshold plasma formation energy (1 μJ for our experimental conditions). The colored dotted lines show the pressures at which the phase transition is observed under static conditions. (**b**) An example of the shock wave diameter evolution registered in shadow photography experiments and the pressure on the shock wavefront. (**c**) Example of shock wave shadow photographs.

The pressures achieved at the wavefront of the laser-induced shock wave could overcome the threshold pressure. We performed shadow photography measurements to retrieve the shock wave pressure (see “Methods” section). We obtained shadow photographs (see Fig. 2c) for different energies (the threshold energy of plasma formation is about 1 μJ). In the shadow photographs, the shock wave is observed as a divergent spherical wave (see Fig. 2a). We varied the energy from 1 μJ up to 14 μJ. By approximating the dependence of the shock wave diameter on time (see the inset in Fig. 2a) with an exponentially decaying function, we obtained the dependence of the shock wave velocity on energy. Then, using shock adiabat (see “Methods” section), we restored the pressures for given laser energies (see Fig. 2b). In experiments, we achieved pressure higher than 50 GPa with damping rate ~ 10 ns (on the moving shock front). Initially the shock wave velocity under such conditions is about 12 km/s (for 4 μJ pump pulse).

According to the phase diagram of MgF_2_^22^, the pressures achieved in the experiments are higher than the threshold values for low-temperature phase transitions in MgF_2_ (Pnam, Pa-3, Pnmn) under static conditions. These phase transitions are characterized by the change of the lattice symmetry due to the decrease of interatomic distances. Under these conditions, the interatomic potential changes abruptly, determining new atomic positions. The presented in Fig. 2 threshold pressure is determined under static conditions when the new phase can exist infinitely in time. However, the shock wave propagation induces ultrafast (with a characteristic time of a picosecond) change in pressure, and the achieved pressures could not serve as a trustworthy indicator of a phase transition^27^.

Moreover, in the dynamical case, the phase transition is metastable and could be reversible, returning to its original state after the passage of the shock wave. If the new phase exists, it has to affect the macroscopic properties of the medium. To verify the possibility of phase transitions under laser impact, we retrieve the phonon spectrum of MgF_2_, which is determined by the lattice symmetry.

**Figure 3:**
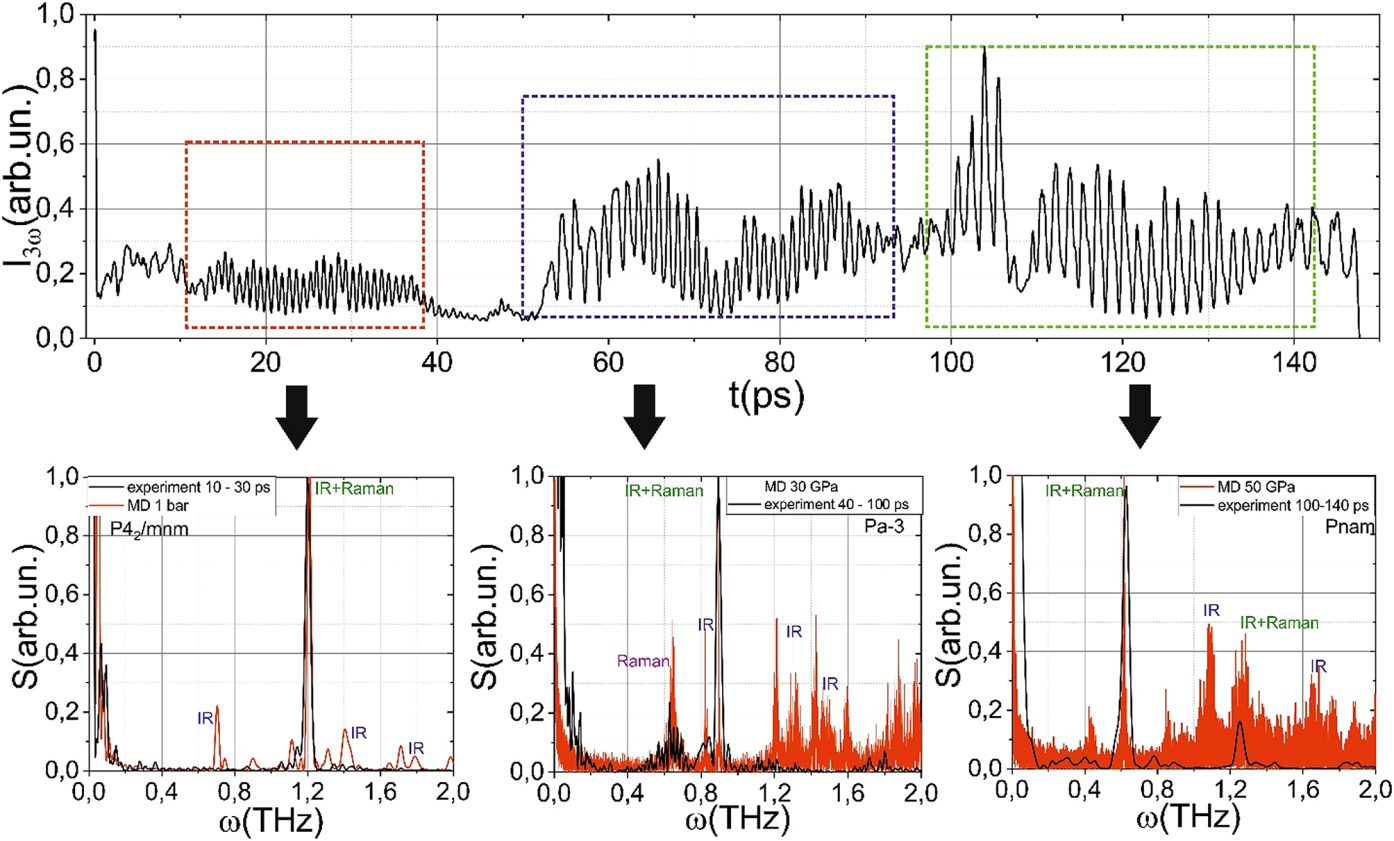
Dependence of the third harmonic signal on the time delay between the pump and probe pulses. Bottom black lines show the spectra of this signal in time windows, marked with a colored dotted line. The red lines show the phonon spectra (multiplied by the ω^−3^ function), obtained in numerical simulations for pressures of 1 bar, 30, and 50 GPa. The modes in the simulated spectra are marked if they are Raman or IR active.

To retrieve the phonon spectrum, we recorded the dependence of the third harmonic (TH) signal on the time delay between the pump and probe laser pulses (see Fig. 3). The phonons, induced by the displacement of ions in lattice, drive the modulation of the third-order susceptibility χ^(3)^^28^:

1 $$\chi^{(3)} = \chi_{0}^{(3)} + \left( {\frac{{\partial \chi^{(3)} }}{\partial Q}} \right)Q,$$

where χ_0_^(3)^ is the third-order susceptibility of the unperturbed sample, *Q* is the displacement of atoms in a lattice and $\left( {\frac{{\partial \chi^{(3)} }}{\partial Q}} \right)$ is the polarizability tensor. The efficiency of third harmonic generation (THG) process is proportional to:

2 $$\eta _{{3\omega }} \sim \left( {\chi ^{{(3)}} } \right)^{2} /\Delta n^{{2.6}},$$

where ∆n is the refractive index difference between the third harmonic and the fundamental pulse^29^. Moreover, this refractive index difference between (without plasma impact) does not depend on phonon frequency $\Delta n = \Delta n_{0} + \left[ {n_{2} \left( {\lambda_{1} } \right) - \,n_{2} \left( {\lambda_{3} } \right)} \right] \times I$, where *I* is laser pulse intensity, ∆*n*_*o*_ is phase mismatch of unaffected MgF_2_, *n*_2_ is nonlinear refractive index *λ*_1_ = 1240 nm and *λ*_3_ = 413 nm. Thereby the third harmonic signal would be modulated on the phonon frequency, and the permitted phonon modes would be determined by $\left( {\frac{{\partial \chi^{(3)} }}{\partial Q}} \right)$ tensor.

Compared with the experiments on the phonon generation in Sb_2_^30^, the peak fluences (~ 10^4^ J/cm^2^) and intensities (~ 10^13^ W/cm^2^) in our experiments are about four orders higher, leading to the electron plasma ignition. According to the femtosecond laser-matter interaction regime, laser pulse energy cannot be transferred to the lattice directly, and phonons are generated with a delay of ~ 10 ps. As a result, the laser-induced plasma acts as a broadband excitation (close to delta function) for coherent phonons. The delay for the coherent phonons generation corresponds to the characteristic time of electron–phonon coupling^28^. For stationary case it could be estimated as $\frac{1}{{\tau_{k} }} \approx \frac{{\left| \xi \right|^{2} m_{c} \theta k}}{{\pi \rho c_{s}^{2} \hbar^{3} }}$, where *ξ* is deformation potential constant, m_c_ is the effective mass of the electron, *θ* = *k*_*b*_*T* (*T* is the crystal lattice temperature, *k*_*b*_ is Boltzmann constant), k is the wave vector of electrons in the plasma, *ρ* is the MgF_2_ density and *c*_*s*_ is the speed of sound. In our estimation, we used the following parameters |*ξ* |~ 11 eV (an order of band gap), *m*_*c*_ ~ 0.5 *m*_*e*_, where *m*_*e*_ is electron mass, T = 300 K, *ρ* = 3.15 g/cm^3^, *c*_*s*_ = 3.5 km/s, the wavevector of the electrons was estimated from the kinetic energy estimate of 4 eV^28^. Under such parameters, the energy transport time from electrons to phonons could be estimated as 6 ps.

There is also a nonzero component in the third harmonic intensity on time; it could be caused by such processes as shock wave generation (an increase of density on the shock wavefront will lead to the change in χ^(3)^ and third harmonic generation) and micromodification formation (decrease of density in the center will lead to the change in χ^(3)^ and third harmonic generation). The shock wave will leave the laser spot (~ 2 μm radius) in about 150 ps, which corresponds to the disappearance of coherent phonons.

In the pump-probe experiments (see “Methods” section), the energy of the pump pulse was about 2 μJ, which equals about two thresholds of plasma formation. The energy of the probe pulse was about 100 nJ, more than an order less than the pump energy. The third harmonic signal could be divided into three regions over time (see Fig. 3): 10–40 ps, 50–90 ps, and 100–140 ps. We performed the spectral analysis of these regions. In the first region, the signal at a frequency of 1.2 THz is distinguished, which, with an increase in the time delay, changes its frequency, first by 0.9 THz (second region), and then down to 0.8 THz (third region). In addition, novel spectral components start to appear in the spectrum.

The abrupt change of the spectrum results from the change in the lattice symmetry. If the distance between the atoms changes monotonically, the frequency of the phonon mode will change similarly. In contrast, we obtain a “jump” in the frequency. The jump could only result from the phase transition that leads to the change of the lattice symmetry, which determines permitted phonon modes. However, due to the limits of the method (recorded spectrum lies in the range up to 4 THz), the spectrum itself does not give direct information about phase transitions and achieved pressures.

Interestingly, the obtained modes are limited by 4 THz. That is followed by the generation mechanism, namely displacive excitation of THz phonons (DECP)^31^. Following the approach, the displacement of atoms Q has an inversive quadratic dependence on phonon frequency (ω) $Q\sim 1/\omega^{2}$. On the other hand, $\left( {\frac{{\partial \chi^{(3)} }}{\partial Q}} \right)$ tensor has a square root dependence on phonon frequency $\left( {\frac{{\partial \chi^{(3)} }}{\partial Q}} \right)\sim \sqrt \omega$^32^. Thereby the efficiency of the third harmonic generation has an inverse cubic dependence on frequency:

3 $$\eta_{3\omega } \sim \omega^{ - 3} .$$

Following Eq. (3), the phonon spectrum, retrieved from the molecular dynamics (MD), was normalized on the ω^−3^ function. Interestingly, in the framework of the alternative approach, describing the phonon generation mechanism of impulsive stimulated Raman scattering (ISRS)^33^, the displacement of atoms is inversely proportional to frequency *Q* ~ *1*/ω, but we obtained the inverse cubic dependence that corresponds to a DECP mechanism of phonon excitation. Comparing results of numerical simulation of the phonon spectrum (considering that the intensity of the third harmonic signal is inversely proportional to the cube of the vibration frequency that corresponds to the best coincidence) with experimental data allows us to estimate achieved pressures and identify the phases of MgF_2_ during laser impact (see Fig. 3). The first region (10–40 ps) corresponds to the P4_2_/mnm phase and atmospheric pressure, the second region (50–90 ps) to Pa-3 phase (30 GPa), and the third region (100–140 ps) to Pnam (50 GPa), respectively. Thus, it can be argued that the change in the characteristic modes of phonon vibrations, observed experimentally under femtosecond laser irradiation is caused by a rearrangement of the crystal lattice.

On time scales up to about 40 ps, the crystal lattice still corresponds to an unexcited medium. Then, the amplitude of the phonon vibrations drops significantly. Further, the amplitude of the TH increases, and the fundamental frequency shifts from 1.2 to 0.9 THz. As a visual analogy of the process, one can imagine the two-dimensional array of nonlinear coupled oscillators. After external force is applied, they will start to oscillate on eigenfrequencies. Then the part of oscillators abruptly changes its eigenfrequency; moreover, the fraction of oscillators at the new frequency increases (due to the phase transition). Under such a process, the amplitude of oscillations will decrease because the energy is not dissipated after a short period, the oscillations on the new frequency will re-appear. The characteristic time of starting a new oscillation should correspond to phonon–phonon scattering time ~ 10 ps^34^. A similar process occurs when the frequency shifts from 0.9 to 0.65 THz. Thereby we can claim that after 50 ps after laser-plasma excitation, a phase transition P4_2_/mnm ⇒ Pa-3 occurs. The atoms' shift during phase transition is presented in Fig. 3. This phase is metastable and exists during about 50 ps, after which there occurs phase transition Pa-3 ⇒ Pnam. The last phase also exists over about 50 ps, after which the crystal lattice is finally destroyed (the micromodification is formed), and the third harmonic signal disappears accordingly. An increase in the laser pulse energy (up to 4 μJ) leads to the more rapid destruction of the material and a decrease in the number of observed phases is taken place.

Remarkably, we did not observe phonon spectrum specific for the Pnmn phase. It could be caused by a relatively narrow range of pressures, where this phase is stable (see Fig. 2a). The spectrum retrieved from the MD corresponds to the phonon density of states of MgF_2_. This spectrum indicates all possible frequencies of atom oscillations, and some of them could not be permitted due to zero components of $\left( {\frac{{\partial \chi^{(3)} }}{\partial Q}} \right)$ the tensor. The MD could also give information about Raman and IR activity of the obtained modes. We calculated the autocorrelation functions of dipole moments (their projections on cartesian coordinates). If a vibration changes, the molecular dipole moment (d), it is IR-active. If it leads to the change in the polarizability, it is Raman active (for example, d_x_^2^ + d_y_^2^ and z or xy components are nonzero). We marked the IR and Raman activity of the phonon in Fig. 3. In the experiment, we could only observe phonons that are Raman-active. The analysis of the diagonal components of observed modes shows that probably 1.2 THz mode (10–40 ps) is A_1g_ (diagonal components of Raman tensor a, a, b), the mode with 0.9 THz is B_1g_ (40–100 ps), and 0.65 THz (100–140 ps) is E_g_ mode. The performed numerical simulation was performed at 300 K and did not consider the possible increase of pressure caused by the laser heat of the lattice. Nevertheless, there is a good coincidence between the simulated and experimental data. That could be caused by low thermodiffusion rates (thermodiffusion time ~ 1–10 μs); thereby, the high temperature is observed only in the area of micromodification and does not influence phase transitions.

**Figure 4:**
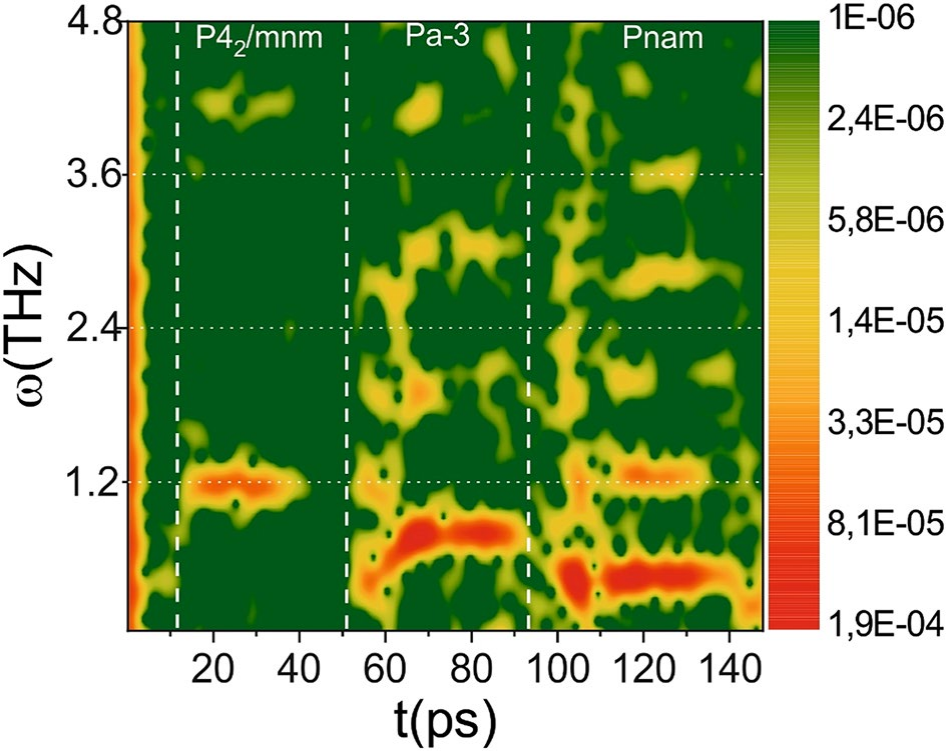
Heat map of the spectrum of phonon vibrations obtained as Fourier transform of the third harmonic signal evolution with a sliding time window. Logarithmic scale.

Applying Fourier analysis with a sliding time window (~ 0.1 ps) to analyze the time signal of the third harmonic, it is possible to visualize the temporal dynamics of phase transitions more clearly (see Fig. 4). The phase transition P4_2_/mnm ⇒ Pa-3 is accompanied by a smooth increase in frequency. The smooth decrease in frequency (with time delays of more than 140 ps) precedes the formation of micromodification, which corresponds to the lattice destruction. It is also worth noting that the spectrum of phonon vibrations changes abruptly in other cases. At phase transitions, a jump-like change in the spectrum is probably caused by the fact that the system’s symmetry changes during phase transitions. With a “new” crystal lattice, “old” vibrations become impossible, leading to a redistribution of energy between phonon modes with a close frequency, similar to the process observed in Ref.^35^. The apparent increase in the signal amplitude in the time dependence is caused by the fact that at higher pressure, the phonon oscillations have a lower frequency, which means that the third harmonic signal will increase.

## Conclusion

To sum up, with the help of the third harmonic-based pump–probe technique and molecular dynamics simulation, we reconstructed the dynamics of laser-plasma induced phase transitions in magnesium fluoride. First, the tightly focused femtosecond laser pulse generates non-equilibrium electron plasma. Once after its relaxation (transferring the energy to the lattice), the coherent phonons at the 1.2 THz frequency are excited. The phonon spectrum at the initial stage of energy transfer processes corresponds to the unperturbed MgF_2_ sample. Then due to a sub-TPa shock wave generation, a cascade of picosecond phase transitions P4_2_/mnm ⇒ Pa-3 ⇒ Pnam occurs. The transition between phases has the characteristic time of 5–10 ps. The phase lifetime is on the order of 40–60 ps; after 140 ps, lattice disordering is observed, and the coherent phonons disappear. In addition, phonon density of states, simulated by molecular dynamics, together with third-harmonic time-resolved spectra prove that laser-excited phonons in a bulk of dielectrics are generated by displacive excitation (DECP) mechanism under plasma mediated conditions.

## Methods

### Pump–probe third-harmonic technique

To retrieve the dynamics of the phase transitions, we used the third harmonic-based pump–probe technique. Under tight focusing of laser radiation inside the unperturbed isotropic medium, the efficiency of third-harmonic generation equals to zero. However, any inhomogeneities violated the destructive interference. The coherent phonons (atoms oscillations) act as inhomogeneities in our case. It should be noted that the intensity of the third harmonic signal, recorded in the experiment, is proportional to ω^−3^ (see above), where ω is the phonon frequency is 10 Hz). In the experiments, we used Cr: Forsterite laser system (central wavelength is 1240 nm, pulse duration is about 100 fs, repetition rate is 10 Hz). Using Michelson interferometer with one moving mirror, we divided the initial laser pulse onto pump and probe pulses (both pulses have the same wavelength)^21^.

The pump pulse (energy is varied from 1 μJ up to 14 μJ) generates plasma (electron density ~ 10^18^ cm^−3^), while the second acts as a probe (laser energy is 100 nJ). Both pulses are tightly focused into the bulk of magnesium fluoride using a Thorlabs A240 TM lens (NA = 0.5 f = 8 mm). The resulting fluence and intensity inside the sample are ~ 10^4^ J/cm^2^ and 10^13^ W/cm^2^ respectively. The laser radiation is focused into a spot with a diameter of ~ 4 μm. The sample was automatically moved using motorized translation stages synchronously to the laser pulse repetition rate. Under such experimental conditions, a single-pulse interaction regime with the sample was established. The energy of the pump and probe pulses is controlled using calibrated photodetectors. A Michelson interferometer was assembled to change the time delay between the laser pulses. By varying the length of one interferometer arm, we changed the time delay between the pump and probe pulses. In one of the arms of the interferometer, a quarter-wave plate was placed. A double pass through the plate rotated the polarization by 90 degrees. A polarizer was placed in front of the detection system, adjusted in such a way as to transmit only a probe pulse.

In contrast to the traditional pump-probe schemes, in our experiments, the signal of non-synchronous third harmonic (TH), generated on coherent phonons, was recorded. The TH signal was registered by a photomultiplier tube (Hamamatsu H10722-113). As we have shown in our publications earlier, this technique is much more effective than the traditional one, within which the radiation is recorded at the fundamental wavelength^29,36^. A detailed description of the experimental setup can be found elsewhere^29,36,37^.

### Time-resolved shadow photography

Time-resolved shadow photography was used to determine the achieved laser-induced pressure in the bulk of magnesium fluoride. The main aim of the technique is to determine the shock wave velocity that determines shock wave pressure. As in the THG technique, the temporal resolution is achieved by changing the optical path between the pump and probe pulses. Within the framework of this technique, the second harmonic of the Cr:Forsterite laser radiation (620 nm), generated in a type I BBO crystal, was used as a probe pulse. As in the case of probing by the third harmonic, the pump pulse is tightly focused into the sample; however, the probe pulse was preliminarily scattered on the diffusion plate and directed opposite to the pump pulse. As a result, the focusing lens collects the signal from the waist on the CCD camera. The probe pulse passing through the irradiated area undergoes refraction on the changes in the refractive index induced in the sample during the propagation of the laser-induced shock wave, which in shadow photographs looks like dark areas. We determined the shock wave diameter for each time delay and fitted the resulted function as an exponentially decaying function with a dumping parameter α^38^. From the fitting parameters, we determined the shock wave velocity and dumping parameter. Because the pressure and the velocity of the shock wave are uniquely related by the equation of the shock adiabat, then using the velocity of the shock wave at the initial moment (calculated from several shadow photographs taken for different time delays), it is possible to estimate the pressure of the shock wave.

4 $$p = p_{0} + \rho U_{s} U,$$

5 $$U_{s} = c_{0} + \lambda U$$

where *p* is shock wave pressure, *p*_0_ is the pressure in the unperturbed material, ρ_0_ is density, *U*_*s*_ is shock wave speed, *U* is particle velocity *c*_0_, and λ are empirical constants *c*_*0*_ = 3.5 km/s and λ = 1.56 km/s^39^. Then we calculated the pressure on the shock wavefront for each time delay and pump energy, as it is shown in Fig. 2.

### Molecular dynamics simulations

**Figure 5:**
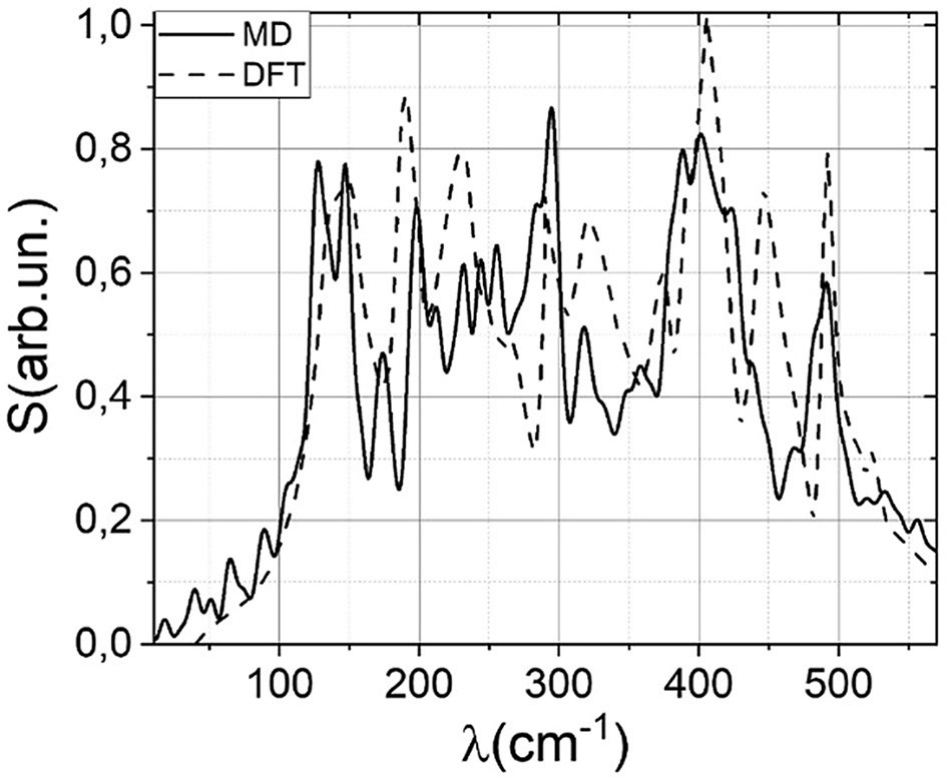
Phonon density of states retrieved from MD simulations (solid line) and reproduced from Ref.^42^.

The LAMMPS software package was used for modeling^40^ the phonon spectrum of MgF_2_. The applied potential for fluorides was presented in the paper^41^. This model is valid up to the pressures of 130 GPa. However, in a numerical experiment, the destruction of the grid was observed at pressures above 100 GPa. Within the simulation framework, one supercell of magnesium fluoride with a size of 100 by 100 per 100 atoms was created. External stationary pressure was applied to each boundary. The simulation was carried out in the n-p-T ensemble. Before the simulation, the system was equilibrated at room temperature and atmospheric pressure (1 bar). Since the phase transition does not occur in a system with ideal periodic boundary conditions, 100 atoms, randomly distributed along with the simulation cell, were removed. They served as seed centers for the new phase. The phonon (vibrational–rotational) spectrum was calculated as follows. First, the velocity autocorrelation function was calculated for 1,000,000 steps (time step 0.1 fs). Then the Fourier transform of the autocorrelation function was performed. The spectrum obtained in this way is the rotational-vibrational spectrum of the test substance. The simulations were performed with a 10 GPa step. The phonon spectra obtained in this way agree with the tabulated data. Figure 5 below demonstrates the comparison of the calculated phonon spectrum with the estimated one from the time-domain density functional theory. We also simulated the instantaneous value of dipole moment (d) and its protections on cartesian coordinates. For this purpose, we added the charge of each atom to the measurement of the autocorrelation function. To determine if the obtained mode is IR or Raman active, we plotted the FFT spectrum of (d, d_x_^2^ + d_y_^2^, d_x_ × d_y,_ and d_z_). If there is a nonzero component in d the mode is IR active and if d_x_^2^ + d_y_^2^ and d_z_, or d_x_ × d_y_ are nonzero than the mode is Raman active.

## Data Availability

Data is available upon reasonable request.
